# 气管支气管成形术后患者的护理体会

**DOI:** 10.3779/j.issn.1009-3419.2014.03.19

**Published:** 2014-03-20

**Authors:** 燕 田, 兰 乌, 桂珍 杨

**Affiliations:** 1 010059 呼和浩特，内蒙古医科大学附属医院护理部 Department of Nursing, Affiliated Hospital of Inner Mongolia Medical College, Hohhot 010059, China; 2 010070 呼和浩特，白塔国际机场有限责任公司航空医疗急救中心 Air Medical Emergency Center, Baita International Airport Co., Ltd., Hohhot 010070, China

气管、支气管成形术是胸外科技术难度较大的术式，不仅最大限度地切除了病变组织，还最大限度地保留了有功能的肺组织。成形术式主要包括气管、隆突、支气管袖状切除成形术，气管、隆突、支气管楔状切除成形术和气管、支气管损伤缝合术三类，具体术式的选择主要依据病变的性质和部位而定^[1]^。气管、隆突、支气管成形术对提高手术切除率、降低手术风险、改善患者术后生活质量、延长存活时间起到了重要作用^[2]^。内蒙古医科大学附属医院2005年5月-2013年4月共完成气管、隆突、支气管成形术71例，效果满意，现将护理体会介绍如下。

## 1 临床资料

### 1.1 一般资料

全组71例，按不同原发疾病分为：①肿瘤性疾病65例，男性54例，女性11例，年龄33岁-76岁，平均63.7岁。主要表现为气短、咳嗽、痰中带血、咳血等。术前行胸部增强CT、脑CT或MRI、腹部彩超或CT、骨扫描评价肿瘤局部及全身转移情况，7例患者术前行PET-CT检查，所有患者均行支气管镜检查。肿瘤位于右肺上叶34例，右主支气管、隆凸8例，左肺上叶12例，左肺下叶11例。病理类型：鳞癌54例，腺癌9例，粘液表皮样癌2例。外伤性气管、支气管损伤6例：男性5例，女性1例，年龄29岁-56岁，平均36.1岁。车祸伤3例，高处坠落2例，重物砸伤1例。伤后至入院时间分别为4 h、6 h、2 d、4 d、4 d、20 d。主要表现为呼吸困难、胸痛、血痰，伴有面、颈、胸部皮下气肿、纵隔气肿、血气胸。所有患者均行胸片和/或胸部CT，术前均经支气管镜检查确诊。确诊为右主支气管断裂2例、右上叶支气管断裂2例、气管下段裂伤及右主支气管断裂1例、左主支气管断裂1例。

### 1.2 手术方法

全组71例患者共完成气管、隆突、支气管成形术72例次，1例外伤性气管下段裂伤及右主支气管断裂，行气管、支气管成形术患者，术后2个月出现右主支气管上叶开口处闭塞，再次手术行右肺上叶袖状切除术。本组14例采用单腔、58例采用双腔气管插管全身麻醉，有5例在术中同时采用经术野插管控制呼吸^[3]^。除1例患者双侧先后开胸（先行左胸小切口开胸松解左肺门，后经右胸后外侧切口行隆突重建）外，余患者均采用后外侧切口进胸。

65例肿瘤患者的手术方式：①右肺上叶袖状切除，右主支气管和中间干支气管端端吻合术29例；②左肺上叶袖状切除，左主支气管和下叶支气管端端吻合术2例；③左肺下叶袖状切除，左上叶支气管与左主支气管端端吻合4例；④右全肺切除合并隆突切除，气管下段与左主支气管端端吻合4例；⑤右肺上叶切除，右主支气管楔形切除成形5例；⑥左肺上叶切除，左主支气管楔形切除成形10例；⑦左下叶切除，左主支气管楔形成形7例。⑧右肺上叶、隆突切除，气管、右中间干支气管、左主支气管重建隆突4例。以上患者同期行肺动脉、上腔静脉、左心房切除成形15例；隆突切除重建术后行颏胸pearson固定8例。

6例外伤患者行气管、支气管成形手术7例次，手术方式：①右主支气管吻合术2例；②右肺上叶支气管吻合术2例；③气管下段裂伤修补及右主支气管吻合术1例，2个月后因右上叶支气管开口处狭窄再次行右肺上叶袖状切除术；④左主支气管吻合术1例。

## 2 结果

71例患者中，无手术死亡。1例双侧开胸的隆突切除重建患者于术后第7天，因支气管吻合口肺动脉瘘致大咳血死亡；1例左肺上叶切除、左主支气管楔形成形术的肿瘤患者术后当日胸腔内出血，再次急诊胸腔镜下探查止血后治愈；1例气管下段裂伤及右主支气管断裂患者，术后2个月出现右主支气管上叶开口处瘢痕狭窄，再次手术行右肺上叶袖状切除后治愈；术后并发肺不张6例，肺部感染3例，呼吸功能衰竭1例，均经对症治疗、抗感染、呼吸机辅助呼吸后治愈。余患者恢复顺利。

## 3 术后护理

### 3.1 一般护理

患者术毕返回术后监护室后，采用去枕平卧位，给予持续低流量吸氧，头稍偏向一侧，防止口腔分泌物或呕吐物误吸，并防止舌后坠。待麻醉清醒、血压平稳后改为上半身抬高的斜坡卧位。持续心电监护，严密观察患者意识、面色、体温、脉搏、呼吸、血压及血氧饱和度的变化。出现心律失常、血压或呼吸异常、低氧血症等异常情况，应及时通知医生。检查输液、引流管、尿管等管道有无移位、脱落，并认真记录出入量。注意根据患者情况控制输液速度，避免短时间内输入大量液体，防止发生肺水肿。带气管插管返回者连接呼吸机辅助呼吸，定时复查动脉血气分析调整呼吸机参数直至停用呼吸机，拔除气管插管。

隆突切除重建术行颏胸pearson固定者，返回监护室后取平卧位，头下垫一软枕，保持头颈前屈位15°-30 °，并注意固定头部，以免患者躁动撕开固定缝线影响气道吻合口。每日对缝线处消毒，防止感染，尤其在夏天，保持前颈部皮肤干燥，防止浸渍。Pearson体位易导致颈部肌肉疲劳，术后经常局部按摩。Pearson体位还影响患者睡眠及日常活动，易产生焦虑、烦躁等情绪，应做好心理护理^[4]^。

### 3.2 胸腔闭式引流管护理

胸腔闭式引流护理是胸外科手术后重要的护理项目，也是观察术后胸腔内情况变化的最直接窗口^[5]^。定时挤压胸管，保证通畅，防止扭曲、受压、脱落。引流瓶每日更换胸液，并严格执行无菌操作。严密观察并记录引流液情况及有无气体逸出。如引流液为浓血性且量大时、或引流瓶内有持续大量气体溢出时，应及时与医生沟通，除外胸腔内出血、支气管胸膜瘘。本组1例左肺上叶切除、左主支气管楔形切除术患者术后当日引流血性液1, 000 mL，再次在手术室胸腔镜探查止血后治愈。

### 3.3 呼吸道护理

术中对肺部的挤压损伤、术后的切口疼痛等常导致开胸患者术后痰液增多、排痰困难，而气管、隆突、支气管成形术患者则由于以下原因使这种情况进一步加重：①呼吸道重建使支气管内膜纤毛排送系统的功能下降；②气道成形术中，术野渗血会使血液流入远端支气管内，增加了痰液的粘稠度；③吻合口出血、水肿影响排痰；④隆突切除重建者，颏胸pearson固定影响咳嗽；⑤隆突切除者正常咳嗽反射消失，痰液容易积聚在气道内，排痰困难^[4]^。可见，气管、隆突、支气管成形术后更易发生呼吸道分泌物积聚、排痰困难，导致肺部并发症和呼吸功能衰竭。因此，术后早期呼吸道护理对手术成败至关重要，必须保持呼吸道通畅，鼓励或帮助患者咳嗽、排痰，减少并预防术后肺不张、肺部感染等并发症的发生。

术后应常规给予雾化吸入，并注意方法，雾化时嘱患者做深而慢的呼吸，深吸气后做短暂的屏息，能使更多药物到达呼吸道远端，增加药物在呼吸道的停留，有效地使雾化颗粒均匀地弥散于粘膜，稀释痰液。定时给患者叩背，协助咳嗽排痰，有条件时也可采用机械振动排痰机，效果更好。

对体质差、咳嗽无力或有肺不张的患者，可先采用鼻导管吸痰。吸痰时应使用橡胶吸痰管，更容易下至气道内。当确认吸痰管抵达气管后迅速吸痰，吸痰过程中如出现缺氧应暂停操作，自吸痰管内直接给氧，反复操作直到痰液吸尽为止。吸痰时动作应轻柔，避免损伤吻合口。本组有6例患者经鼻导管吸痰，其中1例患者体质差、术后咳痰无力、痰液较多，我们将鼻导管留置于气管内12 h，间断多次给患者吸痰获得良好效果。对鼻导管不能插入气管内的患者，协助医师进行床旁纤维支气管镜吸痰，也有部分患者采用床旁气管镜主动吸痰。本组有5例患者行床旁支气管镜吸痰，不仅可吸出各肺段支气管内痰液，同时还可观察气管、支气管吻合口情况。经鼻导管或气管镜吸痰时，均应严密监测氧饱和度，防止因缺氧导致呼吸心跳骤停。

## 4 护理体会

气管、隆突、支气管成形术可最大限度保留有功能的肺组织，提高患者的生活质量，其技术操作也已日趋成熟，但手术难度和风险仍较大，除了术前认真准备，术中耐心仔细操作，术后护理也极为重要，尤其是呼吸道护理。术后早期密切监护及处理、保持呼吸道通畅、维持良好的组织氧供、预防肺部感染等术后并发症、促进吻合口愈合，是保证患者手术成功，术后顺利恢复的重要环节。
